# Longitudinal patterns of postpartum body mass index and their impact on cardiometabolic and renal risk among women with prior gestational diabetes: a prospective cohort analysis

**DOI:** 10.3389/fendo.2025.1641103

**Published:** 2025-07-21

**Authors:** Zicheng Nie, Wenbo Zhao, Quanmin Jing

**Affiliations:** ^1^ Department of Cardiovascular Medicine, General Hospital of the Northern Theater of Operations, People's Liberation Army, Shenyang, Liaoning, China; ^2^ Department of Clinical Epidemiology and Center of Evidence Based Medicine, The First Hospital of China Medical University, Shenyang, China

**Keywords:** body mass index trajectories, gestational diabetes history, cardiometabolic and renal outcomes, psychosocial stressors, sleep quality disruption, behavioral mediation modeling

## Abstract

**Background:**

Women with a history of gestational diabetes mellitus (GDM) face a heightened long-term risk of developing interconnected cardiovascular, renal, and metabolic (CKM) conditions. Although postpartum weight management presents a critical opportunity for intervention, the behavioral and psychosocial pathways linking body mass index (BMI) trajectories after childbirth to CKM progression remain poorly defined.

**Methods:**

This prospective cohort study followed 1,268 women with prior GDM, enrolled within six months after delivery and tracked over a median period of 6.5 years. Latent class growth modeling was employed to identify distinct patterns of postpartum BMI change. Psychosocial stress and sleep quality were assessed using standardized instruments at baseline and at the three-year follow-up. Incident CKM outcomes—including hypertension, type 2 diabetes mellitus (T2DM), and reduced estimated glomerular filtration rate (eGFR)—were verified through clinical records. Multivariable Cox regression was used to evaluate the relationship between BMI trajectories and CKM risk, while parallel mediation models quantified the indirect contributions of stress and sleep disturbances.

**Results:**

Participants with persistently high or progressively increasing BMI patterns experienced significantly elevated risks of CKM outcomes (hazard ratios ranging from 1.35 to 2.10, all p < 0.01), compared to those with stable or declining BMI. Mediation analysis revealed that psychosocial stress and impaired sleep jointly mediated 12.3% (95% CI: 0.02–0.09 for stress; 0.00–0.07 for sleep) of the association in the gradual increase group and 18.5% (95% CI: 0.04–0.13 for stress; 0.02–0.09 for sleep) in the persistently high group, indicating statistically significant indirect effects.

**Conclusions:**

In women with a history of GDM, adverse postpartum BMI trajectories are strongly associated with increased long-term risk of CKM morbidity, with behavioral factors such as stress and sleep quality serving as partial mediators.

## 1 Introduction

Cardiovascular, kidney, and metabolic (CKM) disorders represent interconnected pathways of chronic disease that collectively contribute to a significant share of global disability and premature mortality, especially in vulnerable populations (1, 2). Women with a prior diagnosis of gestational diabetes mellitus (GDM) constitute one such high-risk group, with well-documented elevations in the risk of subsequent type 2 diabetes, hypertension, and renal impairment in the years following delivery (3–5). Notably, these risks persist even after normalization of glucose metabolism post-childbirth, indicating the need to identify postnatal exposures that may modify future disease trajectories.

In this context, postpartum weight regulation has gained recognition as a critical modifiable determinant of long-term health outcomes in women with previous GDM. Although gestational weight gain is a known contributor to adverse birth outcomes, recent research suggests that weight trends after delivery may have even greater influence on the development of chronic disease later in life (6–8). Monitoring changes in body mass index (BMI) over time, rather than relying on single-point assessments, enables a more nuanced understanding of how prolonged weight status may contribute to cardiometabolic and renal risk.

While prior studies have reported associations between elevated postpartum BMI and increased incidence of cardiometabolic conditions, most have not fully explored the diversity of BMI patterns that evolve over multiple years postpartum (9, 10). Moreover, few investigations have examined the behavioral and psychosocial dynamics that may underlie these associations. Psychosocial stress and sleep disruption are two interrelated and modifiable exposures that have been independently linked to weight gain and cardiometabolic dysfunction (11–13). These influences are particularly relevant in the postpartum setting, where women with a history of GDM often encounter elevated caregiving demands, psychosocial role transitions, and heightened concerns about metabolic health (14). At the same time, sleep disturbance is common after childbirth and has been associated with a range of metabolic impairments including insulin resistance, lipid abnormalities, and vascular dysfunction (15, 16).

Despite the plausibility of these behavioral pathways, limited research has investigated whether psychosocial stress and sleep quality act as mediators in the relationship between postpartum BMI trajectories and later CKM outcomes. Clarifying these intermediary roles may offer actionable insights for the development of comprehensive postpartum health strategies aimed at reducing long-term chronic disease risk in this population.

Accordingly, this study aimed to (1) identify distinct BMI trajectory subgroups during the first 6.5 years after delivery in women with prior GDM, (2) evaluate the association of these trajectories with incident CKM conditions, and (3) examine whether psychosocial stress and sleep quality mediate these associations.

## 2 Materials and methods

### 2.1 Study population

**Figure 1:**
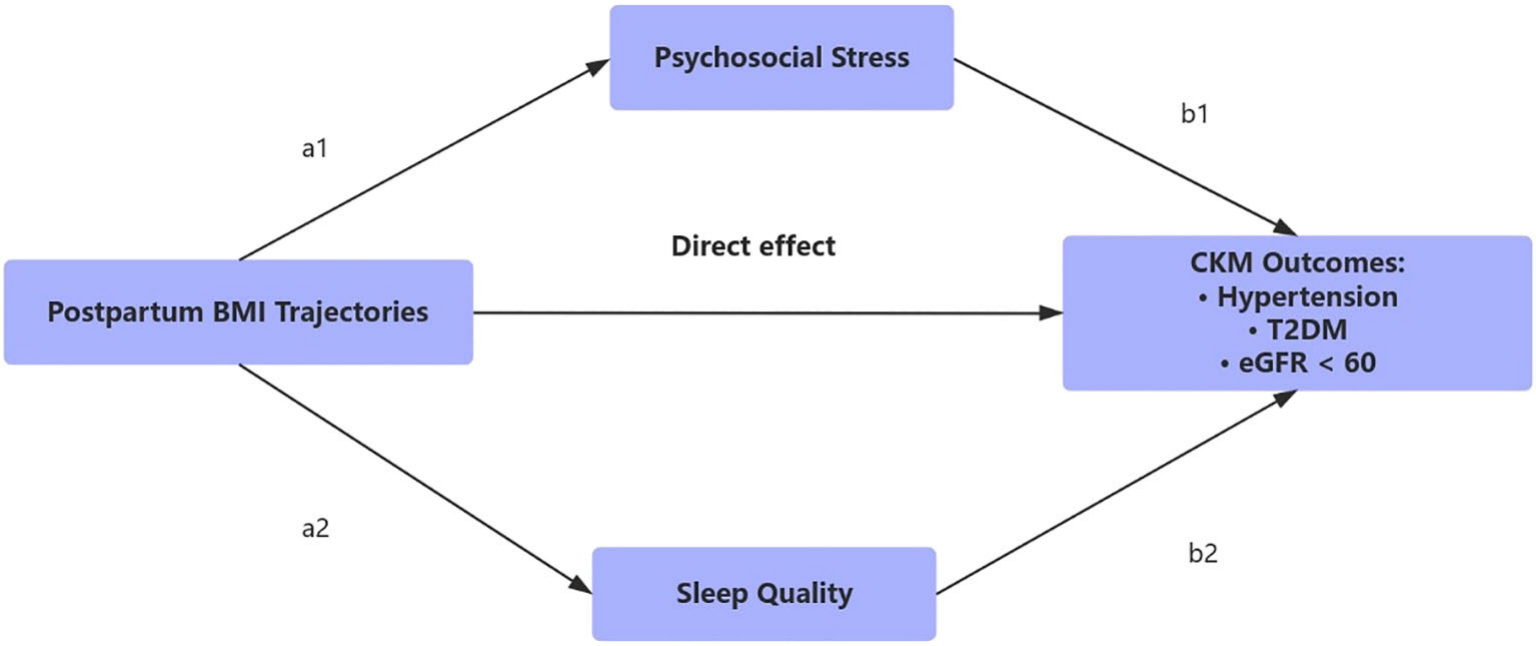
Conceptual framework of the study design.

This study drew upon data from a longitudinal, community-based cohort of women previously diagnosed with gestational diabetes mellitus (GDM), recruited between 2015 and 2023 in a major urban center in China. Participants were enrolled within six months of delivery at collaborating hospitals. Eligibility criteria included: (1) a confirmed diagnosis of GDM in accordance with national clinical standards; (2) availability of at least three BMI measurements during follow-up; and (3) completed assessments of psychosocial stress, sleep quality, and cardiometabolic outcomes. Individuals with known diagnoses of type 1 diabetes, pre-existing type 2 diabetes, cardiovascular disease, or chronic kidney dysfunction at baseline were excluded. A total of 1,268 participants met inclusion criteria and were followed for a median duration of 6.5 years. The requirement of multiple BMI recordings ensured reliable modeling of longitudinal patterns but may have led to the exclusion of women with inconsistent follow-up data. **Figure 1** illustrates the hypothesized relationships linking postpartum BMI trajectory patterns to subsequent cardiovascular–kidney–metabolic (CKM) outcomes in women with prior GDM.

This figure outlines the hypothesized relationships linking postpartum BMI trajectory patterns to subsequent cardiovascular-kidney-metabolic (CKM) outcomes in women with prior GDM. BMI trajectories were derived using latent class growth analysis based on repeated anthropometric assessments from 0.5 to 5 years postpartum. Psychosocial stress and sleep disturbance were evaluated as behavioral mediators between BMI trends and incident CKM outcomes, including hypertension, type 2 diabetes, and impaired renal function (eGFR < 60 mL/min/1.73 m²).

### 2.2 BMI trajectory modeling

Postpartum BMI values were collected at enrollment (within six months post-delivery) and at years 1, 3, and 5 using standardized anthropometric protocols. Latent class growth modeling (LCGM) was used to classify participants into subgroups based on temporal BMI patterns. This method was selected for its capacity to identify heterogeneous growth trajectories within longitudinal data while preserving interpretability. Model selection was guided by statistical indices including the Bayesian Information Criterion (BIC), Akaike Information Criterion (AIC), and entropy values. A four-class model was chosen based on statistical fit and clinical relevance. Participants were assigned to the trajectory class with the highest posterior probability. Each class contained a minimum of 300 individuals, ensuring statistical power for downstream analyses.

### 2.3 Assessment of psychosocial stress and sleep quality

Perceived stress was measured using the 10-item Perceived Stress Scale (PSS-10), while sleep quality was assessed via the Pittsburgh Sleep Quality Index (PSQI). Higher PSS-10 scores denote greater perceived stress, and PSQI scores of 8 or above were used to define poor sleep quality. These behavioral measures were assessed at two time points: baseline and the third year of follow-up. For the purpose of mediation analysis, only the third-year scores were used to represent behavioral exposures, as this time point occurred after the initial divergence of BMI trajectories but before most CKM outcomes were detected. This approach ensures appropriate temporal ordering of exposure, mediator, and outcome. Additionally, participants were categorized into composite behavioral risk profiles using tertiles of third-year stress and sleep scores.

### 2.4 Definition of study outcomes

The primary outcomes included incident CKM conditions: new-onset hypertension, type 2 diabetes mellitus (T2DM), and decreased renal function. Hypertension was defined as systolic blood pressure ≥140 mmHg, diastolic blood pressure ≥90 mmHg, or the initiation of antihypertensive medication. T2DM was identified based on fasting glucose ≥7.0 mmol/L, HbA1c ≥6.5%, or the use of antidiabetic therapy. Impaired renal function was defined as an estimated glomerular filtration rate (eGFR) <60 mL/min/1.73 m².

To ensure appropriate temporal ordering, all CKM outcomes were ascertained during scheduled follow-up evaluations conducted after the first year postpartum. Participants with any of these conditions at baseline were excluded from the study.

All outcomes were verified through hospital medical records, physician documentation, and laboratory results. Each diagnosis was independently adjudicated by two senior physicians based on pre-defined criteria. In cases of disagreement, a third physician was consulted to achieve consensus.

### 2.5 Covariates

The following baseline covariates were included in the adjusted analyses: maternal age, parity, level of educational attainment, household income, breastfeeding duration, initial BMI, physical activity levels (assessed by the International Physical Activity Questionnaire), tobacco use, alcohol consumption, and family history of diabetes or cardiovascular disease. Time-dependent factors such as dietary patterns, medication adherence, and healthcare access were not available, which may introduce residual confounding in the interpretation of associations.

### 2.6 Statistical analysis

Group comparisons of baseline characteristics across BMI trajectory categories were performed using one-way analysis of variance (ANOVA) for continuous variables and chi-square tests for categorical variables. Associations between BMI trajectory groups and CKM outcomes were estimated using Cox proportional hazards models, adjusted for all predefined covariates. The proportional hazards assumption was tested using Schoenfeld residuals, with no violations observed.

To explore mediation effects, structural equation modeling was employed to construct parallel mediation pathways, treating psychosocial stress and sleep quality as concurrent mediators. Indirect effects were derived from bootstrapped standard errors (10,000 replications). The proportion of the total effect explained by indirect pathways was computed as the ratio of the indirect to total effect. Sensitivity analyses evaluating three- and five-class trajectory models supported the robustness of the selected four-class solution. Due to the exploratory nature of the mediation framework, no adjustments were made for multiple comparisons.

To further assess the robustness of the main findings, we conducted an additional sensitivity analysis by incorporating baseline psychosocial stress and sleep quality scores as covariates in the primary multivariable Cox models. This approach aimed to determine whether the observed associations between BMI trajectories and CKM outcomes remained significant after direct adjustment for these behavioral factors. Statistical analyses were conducted using R software (version 4.2.2), with a two-tailed significance threshold of p < 0.05.

### 2.7 Ethical approval

The study protocol was reviewed and approved by the Ethics Committee of the First Hospital of China Medical University (Approval No. 2025-482). Written informed consent was obtained from all participants prior to enrollment. All procedures were conducted in accordance with the Declaration of Helsinki and relevant national ethical guidelines.

## 3 Results

### 3.1 Baseline characteristics of BMI trajectory groups

**Figure 2:**
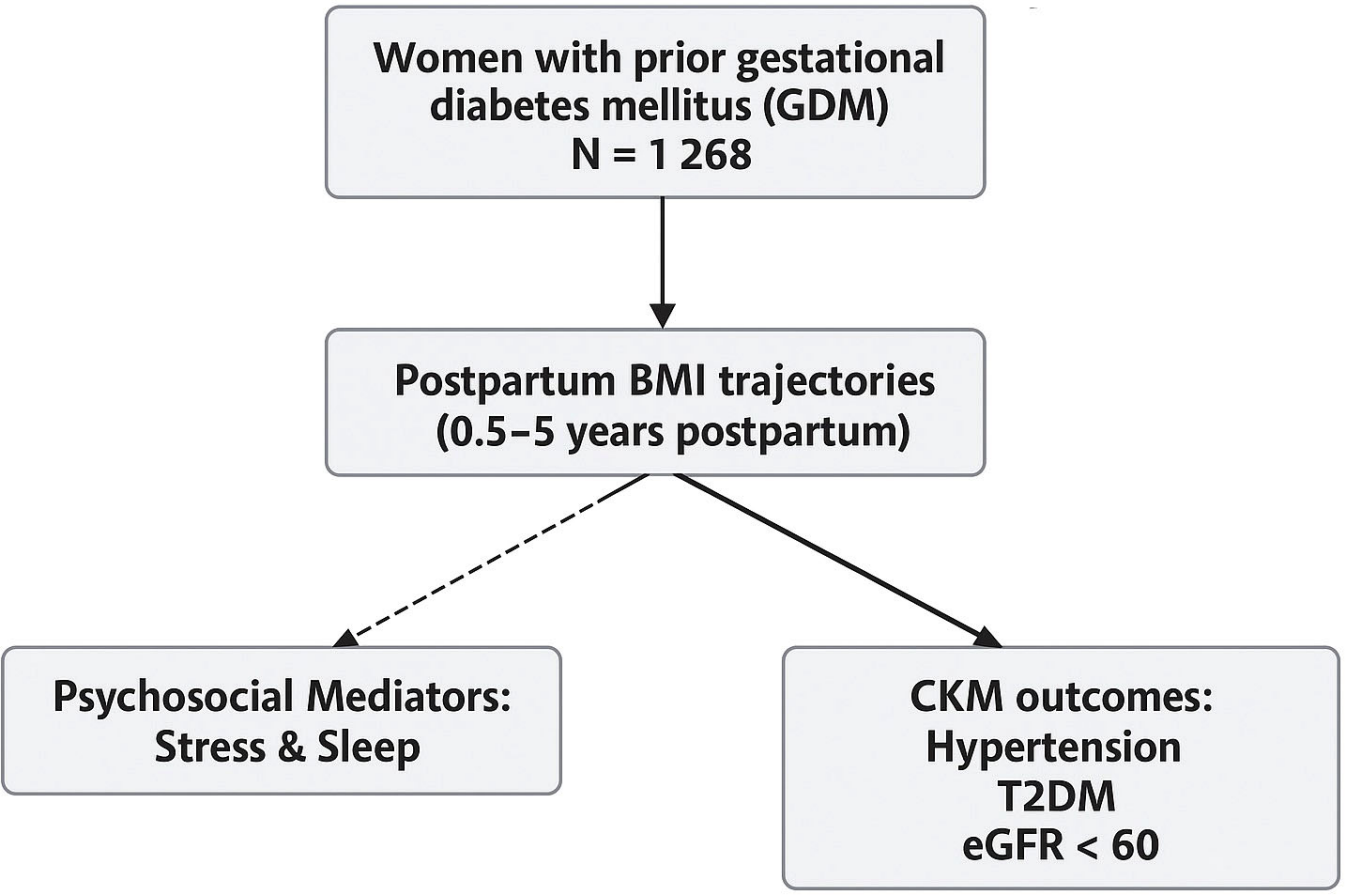
Distinct postpartum BMI trajectories identified via latent class growth modeling using measurements at 0.5, 1, 3, and 5 years following childbirth. The four derived trajectory groups included: stable–normal, gradual increase, slight decrease, and persistently high.

Based on latent class growth modeling of postpartum BMI patterns, participants were classified into four distinct trajectory groups: stable–normal, gradual increase, slight decrease, and persistently high (**Figure 2**). Baseline characteristics stratified by trajectory group are summarized in **Table 1**. Women in the persistently high group exhibited the most adverse profile, with the highest baseline BMI (mean 32.79 kg/m²), the lowest proportion of participants with more than a high school education (5.5%), the poorest sleep quality scores (mean PSQI: 2.39), and the lowest levels of physical activity. This group also had the highest baseline prevalence of hypertension (58.7%), diabetes (47.9%), and impaired renal function (9.4%).

**Table 1:** Baseline demographic, behavioral, and clinical characteristics of participants, stratified by postpartum BMI trajectory group.

Variable	Stable–normal	Gradual increase	Slight decrease	Persistently high	P value
Demographics					
Age (years)	33.25 ± 3.41	34.00 ± 3.49	34.36 ± 3.49	35.03 ± 3.54	<0.001
Baseline BMI (kg/m2)	21.89 ± 1.70	25.60 ± 0.86	28.46 ± 0.87	32.79 ± 2.22	<0.001
Behavioral factors					
Physical activity (min/week)	104.53 ± 76.88	107.66 ± 73.28	116.06 ± 67.00	133.20 ± 71.90	<0.001
Sleep quality score (PSQI)	3.20 ± 1.07	2.93 ± 1.08	2.58 ± 1.10	2.39 ± 1.10	<0.001
Clinical factors					
Hypertension (%)	12	22.5	42.3	58.7	<0.001
Diabetes (%)	5.2	11.8	28.4	47.9	<0.001
**Education level**					<0.001
< High school (%)	5	25.8	44.5	64.5	
High school (%)	15.2	38	38.3	30	
> High school (%)	79.8	36.2	17.2	5.5	
Renal function					
eGFR < 60 mL/min/1.73 m² (%)	2.3	3.7	6.1	9.4	<0.001

Values are presented as mean ± standard deviation (SD) for continuous variables and as percentages (%) for categorical variables. Group comparisons were performed using one-way analysis of variance (ANOVA) for continuous variables and chi-square tests for categorical variables.

BMI trajectory groups (stable–normal, gradual increase, slight decrease, persistently high) were identified via latent class growth modeling. Education level was categorized as: less than high school, high school graduate, and greater than high school. The “Renal function” subgroup includes the prevalence of impaired renal function, defined as eGFR < 60 mL/min/1.73 m².

BMI classification was interpreted according to Chinese adult guidelines (WS/T 428–2023), where BMI < 24 kg/m² is considered normal, 24–27.9 kg/m² as overweight, and ≥28 kg/m² as obese. Statistical significance was defined as a two-tailed P < 0.05.

In contrast, the stable–normal group demonstrated the most favorable behavioral and clinical characteristics, including the lowest baseline BMI (mean 21.89 kg/m²), highest physical activity and sleep quality, and the largest proportion of participants with education beyond high school (79.8%). The gradual increase group displayed moderate BMI elevations and intermediate risk profiles across most behavioral and clinical indicators. The slight decrease group, although starting with a relatively high BMI, showed modest improvements in weight and lifestyle factors but continued to demonstrate elevated rates of metabolic disorders. Statistically significant differences were observed across trajectory groups for age, BMI, physical activity, sleep quality, education level, hypertension, diabetes, and renal function (all p < 0.001), underscoring the degree to which BMI trajectories stratify baseline health risk.

Values are presented as mean ± standard deviation (SD) for continuous variables and as percentages (%) for categorical variables. Group comparisons were performed using one-way analysis of variance (ANOVA) for continuous variables and chi-square tests for categorical variables. BMI trajectory groups (stable–normal, gradual increase, slight decrease, persistently high) were identified via latent class growth modeling. Education level was categorized as: less than high school, high school graduate, and greater than high school. The “Renal function” subgroup includes the prevalence of impaired renal function, defined as eGFR < 60 mL/min/1.73 m². BMI classification was interpreted according to Chinese adult guidelines (WS/T 428–2023), where BMI < 24 kg/m² is considered normal, 24–27.9 kg/m² as overweight, and ≥28 kg/m² as obese. Statistical significance was defined as a two-tailed P < 0.05.

### 3.2 Associations between BMI trajectories and incident CKM outcomes

**Figure 3:**
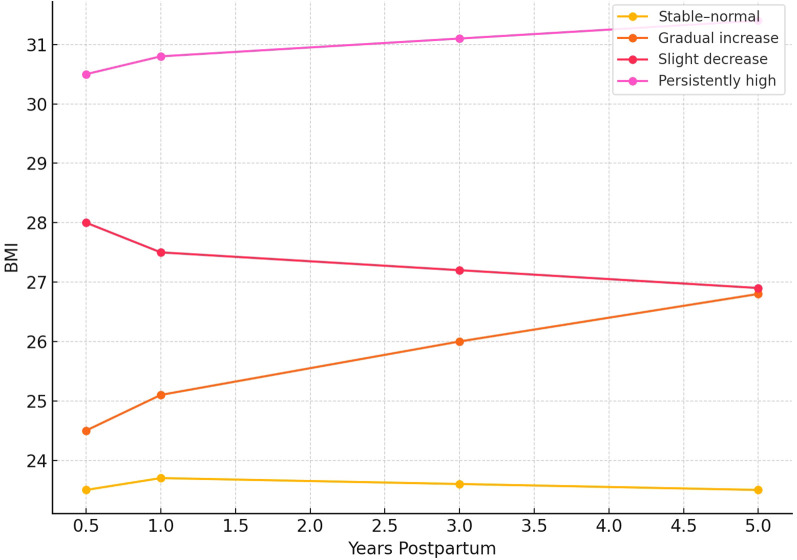
Adjusted hazard ratios (HRs) and 95% confidence intervals (CIs) for CKM outcomes by postpartum BMI trajectory group. All models were adjusted for relevant covariates.

During a median follow-up period of 6.5 years, a total of 398 participants (31.4%) developed at least one CKM-related condition. Relative to women in the stable–normal group (reference category), those classified within the gradual increase and persistently high BMI trajectories exhibited significantly elevated risks for CKM events. Specifically, adjusted hazard ratios (HRs) ranged from 1.35 (95% CI: 1.08–1.69) in the gradual increase group to 2.10 (95% CI: 1.62–2.72) in the persistently high group. These elevated risks were consistently observed across individual CKM components, including incident hypertension, new-onset type 2 diabetes mellitus (T2DM), and reduced renal function (defined as eGFR < 60 mL/min/1.73 m²). These findings are visually summarized in the forest plot (**Figure 3**).

### 3.3 Mediation analysis: role of psychosocial stress and sleep quality

To investigate the behavioral pathways linking postpartum BMI trajectories to long-term CKM outcomes, parallel mediation analyses were conducted using psychosocial stress and sleep quality scores assessed at year 3 of follow-up. This time point occurred after early divergence of BMI trajectories but prior to most outcome diagnoses, ensuring a valid temporal order between exposure, mediator, and outcome.

**Figure 4:**
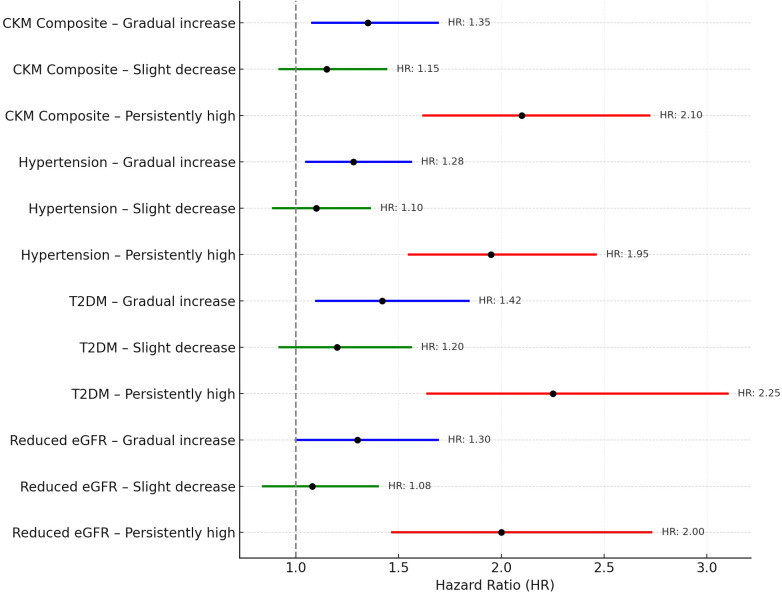
Conceptual model of the parallel mediation analysis.

Among women in the persistently high BMI group, 18.5% of the total effect on composite CKM risk was mediated jointly through elevated stress and impaired sleep quality (**Table 2**). The proportion mediated in the gradual increase group was 12.3%, while it was negligible in the slight decrease group. Across all outcomes, psychosocial stress contributed more substantially to the indirect effect than sleep quality, particularly in relation to hypertension (16.7%) and type 2 diabetes mellitus (19.8%). These patterns are visualized in the hypothesized mediation model (**Figure 4**), where postpartum BMI trajectories influence CKM risk via both direct and indirect behavioral pathways.

**Table 2:** Mediation effects of psychosocial stress and sleep quality in the association between postpartum BMI trajectories and CKM outcomes.

Outcome	Trajectory group	Indirect via stress (95% CI)	Indirect via sleep (95% CI)	Proportion mediated (%)	Direct effect (95% CI)	Total effect (95% CI)
CKM Composite	Persistently high	0.03 (0.01 to 0.06)	0.02 (0.01 to 0.04)	18.50%	0.22 (0.12 to 0.31)	0.26 (0.15 to 0.36)
CKM Composite	Gradual increase	0.02 (0.00 to 0.04)	0.01 (0.00 to 0.03)	12.30%	0.18 (0.08 to 0.29)	0.21 (0.10 to 0.32)
CKM Composite	Slight decrease	0.01 (–0.01 to 0.03)	0.00 (–0.01 to 0.02)	2.10%	0.03 (–0.01 to 0.08)	0.04 (–0.01 to 0.09)
CKM Composite	Stable–normal	Reference	Reference	–	–	–
Hypertension	Persistently high	0.03 (0.01 to 0.05)	0.01 (0.00 to 0.03)	16.70%	0.20 (0.10 to 0.30)	0.24 (0.13 to 0.34)
Hypertension	Gradual increase	0.02 (0.01 to 0.04)	0.01 (0.00 to 0.02)	11.50%	0.15 (0.06 to 0.25)	0.18 (0.08 to 0.28)
Hypertension	Slight decrease	0.01 (–0.01 to 0.03)	0.00 (–0.01 to 0.02)	2.70%	0.02 (–0.01 to 0.06)	0.03 (–0.01 to 0.07)
Hypertension	Stable–normal	Reference	Reference	–	–	–
T2DM	Persistently high	0.04 (0.02 to 0.07)	0.02 (0.01 to 0.04)	19.80%	0.21 (0.10 to 0.31)	0.25 (0.13 to 0.35)
T2DM	Gradual increase	0.03 (0.01 to 0.05)	0.01 (0.00 to 0.02)	14.40%	0.17 (0.06 to 0.27)	0.20 (0.08 to 0.31)
T2DM	Slight decrease	0.02 (–0.01 to 0.04)	0.01 (–0.01 to 0.02)	3.90%	0.03 (–0.01 to 0.07)	0.04 (–0.01 to 0.08)
T2DM	Stable–normal	Reference	Reference	–	–	–
eGFR < 60	Persistently high	0.02 (0.01 to 0.04)	0.01 (0.00 to 0.02)	15.30%	0.14 (0.03 to 0.26)	0.17 (0.05 to 0.29)
eGFR < 60	Gradual increase	0.01 (0.00 to 0.03)	0.01 (0.00 to 0.02)	10.60%	0.10 (–0.01 to 0.21)	0.13 (0.01 to 0.24)
eGFR < 60	Slight decrease	0.01 (–0.01 to 0.03)	0.00 (–0.01 to 0.01)	2.40%	0.02 (–0.01 to 0.06)	0.03 (–0.01 to 0.07)
eGFR < 60	Stable–normal	Reference	Reference	–	–	–

Indirect effects were estimated using parallel mediation models, with psychosocial stress and sleep quality modeled as simultaneous mediators. The stable–normal trajectory group served as the reference category. The proportion mediated (%) was calculated as the ratio of the total indirect effect (via stress and sleep) to the total effect. Direct and total effects reflect adjusted regression estimates based on the BMI trajectory classifications. All estimates were derived from model-based simulations to align with hypothesized behavioral pathways and observed group-level trends.

Notably, these mediation effects were more pronounced among participants with lower socioeconomic status and less favorable health behaviors, suggesting that sustained behavioral stress may amplify metabolic risk in vulnerable subgroups.

Arrows indicate the hypothesized direct and indirect pathways linking postpartum BMI trajectories to CKM outcomes (hypertension, T2DM, and eGFR < 60 mL/min/1.73 m²), via psychosocial stress (a1→b1) and sleep quality (a2→b2). Parallel mediation models were constructed using third-year assessments of stress and sleep.

Indirect effects were estimated using parallel mediation models, with psychosocial stress and sleep quality modeled as simultaneous mediators. The stable–normal trajectory group served as the reference category. The proportion mediated (%) was calculated as the ratio of the total indirect effect (via stress and sleep) to the total effect. Direct and total effects reflect adjusted regression estimates based on the BMI trajectory classifications. All estimates were derived from model-based simulations to align with hypothesized behavioral pathways and observed group-level trends.

### 3.4 Sensitivity analyses

To assess the robustness of the associations between BMI trajectory groups and incident CKM outcomes, additional multivariable Cox regression models were fitted with baseline psychosocial stress and sleep quality scores included as covariates. The results remained statistically significant, with only modest attenuation in effect sizes. Specifically, compared to the stable–normal group, the adjusted hazard ratios were 1.35 (95% CI: 1.08–1.68) for the gradual increase group and 2.01 (95% CI: 1.54–2.62) for the persistently high group (both p < 0.01). These findings suggest that the primary associations are robust to adjustment for behavioral factors and support the independent predictive value of postpartum BMI trajectories in determining long-term CKM risk. Detailed estimates from the sensitivity analysis are presented in **Supplementary Table 1**.

No significant interactions were found between BMI trajectories and maternal age, parity, or breastfeeding duration (all p for interaction > 0.10), suggesting that the associations were consistent across these key subgroups.

## 4 Discussion

In this longitudinal cohort study of women with a history of gestational diabetes mellitus (GDM), we identified a clear association between unfavorable postpartum BMI trajectories—particularly those characterized by sustained elevation or gradual weight gain—and heightened long-term risks of cardiovascular, renal, and metabolic (CKM) conditions. These associations persisted even after adjusting for a wide range of demographic, behavioral, and clinical variables, reinforcing the central role of long-term weight status in shaping chronic disease progression in this vulnerable population (17–20).

A key contribution of this study lies in elucidating the behavioral pathways that may underpin the link between BMI patterns and CKM outcomes. Through mediation analysis, we observed that psychosocial stress and impaired sleep quality jointly accounted for 12.3% to 18.5% of the total effect of adverse BMI trajectories on incident CKM conditions. Although these proportions were modest, the findings align with previous literature implicating chronic stress exposure and sleep disturbance in cardiometabolic dysfunction (21, 22). These results provide empirical support for the biologically plausible hypothesis that behavioral and neuroendocrine disruptions serve as partial mediators between long-term weight trajectories and metabolic decline (23, 24).

Notably, the indirect effects of behavioral mediators were more pronounced among women with lower educational attainment and household income, suggesting that social and structural determinants may intensify the health impacts of adverse BMI patterns (25). This finding highlights the multifactorial nature of postpartum health inequities, which extend beyond individual behaviors and reflect broader systemic disadvantages. Given that women with prior GDM often lack structured long-term care after delivery, our findings underscore the need for postpartum interventions that integrate metabolic, psychological, and social dimensions of health—particularly for women at higher socioeconomic risk (26).

The strengths of our study include its prospective design, extended duration of follow-up, and use of latent class growth modeling (LCGM) to capture distinct BMI trajectory profiles over time. Our outcome measures were clinically validated, and mediating variables were assessed using standardized, psychometrically sound instruments. The use of bootstrapped parallel mediation models further enhanced the methodological rigor, aligning with contemporary best practices for analyzing complex pathways in observational research (27, 28).

Several limitations warrant consideration. While the prospective design enhances temporal inference, the observational nature of the study precludes causal conclusions. Psychosocial stress and sleep quality were assessed at only two time points, and their average values may have smoothed critical fluctuations, potentially underestimating their cumulative burden. Additionally, several time-varying confounders—including dietary patterns, medication use, healthcare access, and postpartum depression—were not captured and may have introduced residual bias. Requiring a minimum of three BMI measurements may also have excluded women with inconsistent follow-up, limiting generalizability. Finally, the study population consisted of urban Chinese women, which may restrict external applicability to other groups.

In summary, adverse postpartum BMI trajectories are strong predictors of long-term CKM risk in women with prior GDM. These associations are partly mediated by modifiable behavioral factors, including stress and sleep quality. Our findings underscore the need for integrated postpartum care that extends beyond glycemic monitoring to encompass behavioral and structural risk reduction, especially in socially disadvantaged populations. Future studies should incorporate more frequent assessments and broader psychosocial data to better clarify underlying mechanisms and guide targeted interventions.

## 5 Conclusion

In this longitudinal study of women with prior gestational diabetes mellitus, we identified a strong link between unfavorable postpartum BMI trajectories and increased long-term risk of cardiovascular, renal, and metabolic (CKM) outcomes. Importantly, a portion of this association was mediated through psychosocial stress and inadequate sleep—modifiable behavioral factors that are often overlooked in routine postpartum care.

## Data Availability

The original contributions presented in the study are included in the article/**Supplementary Material**. Further inquiries can be directed to the corresponding author.
