# Supplementary material for: Longitudinal patterns of postpartum body mass index and their impact on cardiometabolic and renal risk among women with prior gestational diabetes: a prospective cohort analysis
Source: Front Endocrinol (Lausanne). 2025 Jul 21;16:1641103. doi: 10.3389/fendo.2025.1641103 (PMC12318754; doi:10.3389/fendo.2025.1641103)
Supplement: Supplementary file 1 [file Table1.docx]

# Supplementary Table 1

Sensitivity analysis results: Multivariable Cox models with and without adjustment for psychosocial stress and sleep quality.

| BMI Trajectory Group | HR (95% CI), base model | HR (95% CI), adjusted for stress & sleep |
| --- | --- | --- |
| Stable–normal | Reference | Reference |
| Gradual increase | 1.35 (1.08–1.69) | 1.35 (1.08–1.68) |
| Slight decrease | 1.12 (0.87–1.45) | 1.10 (0.86–1.43) |
| Persistently high | 2.10 (1.62–2.72) | 2.01 (1.54–2.62) |
